# A Key Noradrenergic Brainstem-Mesolimbic Circuit: Resilience to Social Stress

**DOI:** 10.1177/2470547019850186

**Published:** 2019-05-17

**Authors:** Hongxing Zhang, Dipesh Chaudhury, Yu Ma, Sarah Montgomery, Jun-Li Cao, Ming-Hu Han

**Affiliations:** 1 Jiangsu Province Key Laboratory of Anesthesiology, Xuzhou Medical University, Xuzhou, China; 2 Jiangsu Province Key Laboratory of Anesthesia and Analgesia Application Technology, Xuzhou Medical University, Xuzhou, China; 3 Division of Science, New York University Abu Dhabi, Saadiyat Island, UAE; 4 Department of Pharmacological Sciences, Icahn School of Medicine at Mount Sinai, New York, NY, USA; 5 Nash Family Department of Neuroscience, Friedman Brain Institute, Icahn School of Medicine at Mount Sinai, New York, NY, USA; 6 Department of Anesthesiology, the Affiliated Hospital of Xuzhou Medical University, Xuzhou, China; 7 Center for Affective Neuroscience, Friedman Brain Institute, Icahn School of Medicine at Mount Sinai, New York, NY, USA

Commentary on: Zhang H, Chaudhury D, Nectow AR, Friedman AK, Zhang S, Juarez B, Liu H, Pfau ML, Aleyasin H, Jiang C, Crumiller M, Calipari ES, Ku SM, Morel C, Tzavaras N, Montgomery SE, He M, Salton SR, Russo SJ, Nestler EJ, Friedman JM, Cao JL, Han MH. α1- and β3-Adrenergic Receptor-Mediated Mesolimbic Homeostatic Plasticity Confers Resilience to Social Stress in Susceptible Mice. Biol Psychiatry. 2019 Feb 1;85(3):226-236. doi: 10.1016/j.biopsych.2018.08.020. Epub 2018 Sep 6. PubMed PMID: 30336931

The adaptive physiological response to acute stress requires the internal milieu of an organism to vary and meet perceived and anticipated demands in the context of a life-threatening situation (i.e., the Fight or Flight Theory). This survival-essential adaptive process is referred to as allostasis in which active homeostasis is rapidly re-established as the acute stressor fades. Incomplete homeostatic rebalancing, especially following repeated stress, leads to long-lasting, maladaptive responses as either psychological and/or physical dysfunctions. Interestingly, some individuals are able to stay phenotypically stable despite exposure to the same severe, prolonged stress. This phenomenon is termed “resilient” to stress. In resilient individuals, additional neural adaptive mechanisms are recruited to re-establish internal homeostasis, allowing them to stay behaviorally stable and cope with future stressors. At present, much less is known about these recruited “resilient” mechanisms in the brain, in contrast to stress-induced pathology in the stress “susceptible” counterpart.

The locus coeruleus (LC), the main source of norepinephrine (NE) in the brain, is comprised of a cluster of NE neurons that are known to be involved in stress and stress-resilience. Many early animal studies have indicated that the LC responds to acute stress and plays an important role in mediating adaptive homeostatic regulation by antagonizing corticortropin-releasing factor.^1^ In human studies, altered LC-NE activity is observed in some patients with psychiatric disorders, such as major depression and post-traumatic stress disorder. Pharmacological blockade of beta-adrenergic receptors in the amygdala prevents the development of aversive memories.^2^ These studies indicate that the LC and its related neural circuits may play an important role in mediating resilience to stress, while an alteration in the responsiveness of the LC to stress may promote resilience to stress. However, more evidence-based research needs to be performed to further explore the defined mechanism.

**Figure 1.**
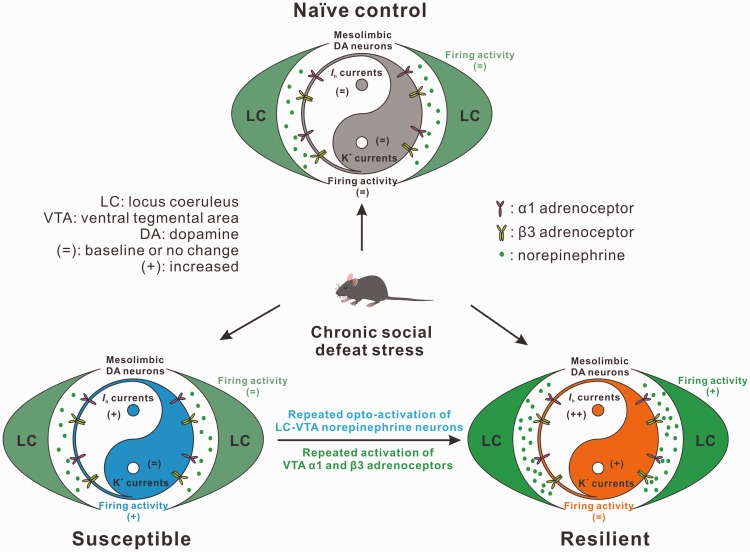
Noradrenergic hyperactivity establishes homeostasis in mesolimbic dopamine neurons to maintain or promote resilience to social stress. Following repeated social defeat stress, mice are segregated into susceptible (depressed) or resilient (non-depressed) sub-populations. Within the resilient group, homeostasis is established by an intrinsic balance of excitatory *I*_h_ and inhibitory K^+^ currents to maintain control-like firing activity in VTA DA neurons that project to the NAc. This current study further expands on this finding by demonstrating that the resilient group exhibits an increase in firing activity of LC neurons that project to the VTA. Repeated optogenetic stimulation induced hyperactivity of the LC-VTA circuit and was sufficient to promote the resilient phenotype in previously defined susceptible mice by re-establishing the aforementioned homeostatic balance in mesolimbic DA neurons. Reversing susceptibility to promote resilience was mediated by VTA α1 and β3 adrenergic receptors.

We recently demonstrated that ventral tegmental area (VTA) dopaminergic neurons projecting to the nucleus accumbens (NAc) constitute a neural circuit in which a resilience-specific homeostasis is established by an intrinsic balance of excitatory *I*_h_ (hyperpolarization-activated cation channel current) and inhibitory voltage-gated potassium (K^+^) channel currents, to maintain control-like neuronal activity and stable behaviors.^3^ More recently, studies from Bruno Giros^4^ and our group^5^ have identified increased activity in the LC-NE neurons projecting to the VTA in resilient mice, following a repeated social stress model for depression. Furthermore, experimentally activating these neurons induced resilience-like behaviors. More importantly, in our circuit-specific molecular profiling study, we identified the α1 and β3 adrenergic receptors as the synaptic relay between the LC-NE system and the VTA-NAc neural circuit, which provide potential translational molecular targets for the development of resilience-promoting antidepressants Figure 1.^5^

Our pharmacological study proceeded by experimentally activating these receptors, infusing a cocktail of their agonists in the VTA. We then observed a re-establishment of intrinsic homeostasis within VTA-NAc DA neurons and resilience-like behaviorial phenotypes in previously defined susceptible mice.^5^ For translational purposes, further studies are needed to examine the role of each receptor independently. Moreover, the LC has a widespread, highly collateralized projection system that innervates the entire neuraxis, including stress/depression-related brain regions such as the medial prefrontal cortex (mPFC) and the amygdala. In our in vitro electrophysiological recordings, we observed a promising increased firing activity in LC-NE neurons that project to the mPFC in resilient mice.^5^ Thus, the LC-NE neurons projecting to other brain targets, including the mPFC, might also hold a potential role in mediating resilience to stress.
